# Development and Characterizations of Novel Aqueous-Based Ceramic Inks for Inkjet Printing

**DOI:** 10.3390/ma16010021

**Published:** 2022-12-21

**Authors:** Haibing Li, Linyu Yang, Feng Li, Qinglong Xian

**Affiliations:** 1 State Market Regulation Technology Innovation Center (Asia Energy Metrologia), Xinjiang Uygur Autonomous Region Research Institute of Measurement and Testing, 188 East Hebei Road, Urumqi 830011, China; 2 School of Physics and Technology, Xinjiang University, Urumqi 830049, China

**Keywords:** ceramic inks, nanoparticle, rheological property, inkjet, printable suspension

## Abstract

Stable rheological properties of ceramic ink are a key requirement for inkjet printing (IJP), which should be satisfied in terms of the Reynolds and Weber numbers. In this paper, the reverse microemulsion was introduced for the synthesis of monodispersed nanosized ceramic powders, and the average size was less than 100 nm. A comparison of two different dispersants, i.e., polyacrylic ammonium (PAANH_4_) and polyacrylic aid (PAA), revealed that the former exerted a good dispersion effect on the ceramic ink. The sedimentation ratio, zeta potential, surface tension, viscosity, and density of the inks were measured, and the Reynolds and Weber numbers, as well as Z value, were calculated. A stable, homogeneous, and high solid loading (20 wt%) ceramic ink could be achieved after aging for a period of 72 h. Finally, the ceramic inks showed the desired printable property in the inkjet printing process. Combining inkjet printing technology with a sintering process, Ni-Mn-O films have the potential to monitor temperature and humidity parameters for intelligent wearable devices.

## 1. Introduction

With the virtue of low cost, low material consumption, and easily changeable digital print patterns, inkjet printing has found numerous applications such as composites and polymers [1,2], network transistors based on semiconductor films [3,4,5], solar cells [6], and wearable electronic devices [7], as well as biological structures for cell interactions and artificial organs [8,9]. The range of ink materials has also been extended to metals and polymers [10,11,12], oxide ceramics [13,14,15,16], and solder pastes for microelectronics’ soldering [17]. Among these materials, with massive research into the inkjet printing of polymers and metals, inkjet printing of ceramics is now the trend of research hotspots. Ceramics are employed in a wide range of applications, owing to their various excellent properties (high mechanical strength, good thermal stability, and so on). Applying conventional technologies, it is a limitation that porous structures with complex geometries are impossible to obtain. Meanwhile, it is different to acquire good quality and precision dimensional in their hardness as well as brittleness. Thus, advanced additional manufacturing combined with ceramic inks becomes a suitable solution. Moreover, IJP has become an essential method for manufacturing advanced ceramics for biomaterials and tissue engineering, e.g., scaffolds for bones and teeth in the medical field.

The practicability and versatility of inkjet printing are attributed to the property of the ink. However, there are several factors that hinder the widespread application of ceramic inks. The first limitation is that the ink must undergo a liquid to solid state transition after printing, which may need further treatment such as solidification and sintering to achieve the designed component. Ceramic inks often have a low loading content, leading to a long drying time and large shrinkage, which can affect the accuracy of the final printed part. The second one requires that the inks be printable so as to satisfy a range of physical properties and to allow repeatable and stable drop formation through the nozzle. The inks should possess good stability, dispersity, and homogeneity, as well as suitable surface tension, viscosity, and density. Recently, an automatized manufacturing of 3D porous aerogel and xerogel networks on semiconducting electrode surfaces via inkjet printing has been reported. More importantly, adjusting the concentration of nanorods within the aqueous ink along with the content of H_2_O_2_ and the optimal ratio of inks and destabilization agent was shown to be a reliable way to enrich the printable properties of the inks [18]. Fu et al. applied an inkjet printing method with silver nanoparticles as the conductive ink to prepare a bimodal sensor that could measure pressure and blending strain simultaneously [19]. Gao et al. [20] presented printable perovskite quantum dot (QD)-based inks using a mixture of dodecane (DOE) and toluene (TOL) solvents, whereby the coffee ring-free and low-roughness perovsike microarrays were successfully deposited onto the PVK (poly-(9-vinylcarbazole)) layer by adjusting the ink formation process. The relationship between the evaporation-driven capillary flow and Marangoni flow as well as the properties of perovskite QD inks were also discussed, but the ink had a very low solid content (15 mg/mL) [20]. Yong et al. reported a stable Y_2_O_3_-stable ZrO (YSZ) jetting for 4 weeks, although containing a very low solid content (5.5 wt%) [21]. Other researchers used the prepared 5–15 wt% YSK ceramic ink, which can be applied by inkjet printing for 3 days [22]. All of the above-mentioned ceramic inks have a relatively low solid content, which is not suitable for the dense ceramic component. It should be noted that the biggest challenge lies to the super stable ceramic inks with a high solid content and relatively suitable viscosity for inkjet printing, as the higher solid content induces an unsuitable viscosity. Through the electrostatic repulsion and the spatial steric hindrance effect, the dispersant mixed with the aqueous solvent and the additive influenced the dispersion property of the ceramic ink. The long-term stability ceramic ink with a well-dispersed ceramic powder was obtained by adjusting the zeta potential. Finally, the as-prepared inks should have printability to meet the demand of the printer device. Specifically, the droplet must not clog the nozzle and show no satellite droplets. However, the solid loading and printability of the inks were not systematically studied, especially for functional ceramic inks.

In view of the above, this work is aimed at developing and fabricating a long-term stable and homogenous aqueous Ni-Mn-O ceramic ink that can be employed for the fabrication of temperature and humidity sensors. Nanoparticles with an average size of 70 nm were synthesized using the reverse microemulsion. According to the defined amount of dispersant and ink formation, ceramic inks with a high solid content of 20 wt% and long-term stability were successfully prepared. The relationships between solid content, viscosity, surface tension, density, and sedimentation ratio, as well as the zeta potential versus the pH value, were systematically discussed, and the actual inkjet printing test verified the ceramic ink printability. Thereby, the printable inks provide a pathway to inkjet printing of functional ceramic materials.

## 2. Materials and Methods

### 2.1. Materials’ Synthesis

A reverse microemulsion method was applied in the present work [23]. Briefly, 30 mL of 0.33 M Mn(NO_3_)_2_·6H_2_O (99%), Ni(NO_3_)_2_·6H_2_O (99%) solution was dropped into deionized water to form a mixture solution. Then, 40 mL of Triton X-100, 60 mL of n-hexanol, and 200 mL of cyclohexane were dissolved in the blend, which was afterwards continuously stirred to form a homogenous microemulsion. After that, 25 mL of NH_3_·H_2_O (25 wt%) was mixed with the microemulsion, and continuous stirring at room temperature was then performed for 6 h. After standing and aging for 24 h, 10 mL of ethanol was added to the above solution to achieve the emulsion breach. The precipitate was centrifuged and filtered and then washed with ethanol and deionized water several times. The obtained material was heated in an oven at 80 °C for 12 h, then calcined at 400 °C for 2 h in a muffle device, and finally ball-milled with polyacrylate ammonium and polyacrylate (solid content of 0.25 wt%, 0.5 wt%, 1 wt%, 1.5 wt%, and 2 wt%) for 12 h. Mn(NO_3_)_2_·6H_2_O (99%) and Ni(NO_3_)_2_·6H_2_O (99%) were obtained from Aladdin. Triton X-100 was purchased from Sinopharm Chemical Reagent Co. Ltd, Beijing, China. n-hexanol and cyclohexane were supplied by Tianjin Baishi Chemical Co. Ltd, Tianjin, China. NH_3_·H_2_O was obtained from Tianjin Fuchen Chemical Co. Ltd, Tianjin, China. All materials were used directly without further purification.

The above powders were blended with deionized water and PEG 400 in proportion and then mixed with different solid loadings (5 wt%, 10 wt%, 15 wt%, 20 wt%, and 25 wt%). The inks were then kept over 72 h in measuring cylinder of 5 mL at room temperature to identify the homogeneous properties.

### 2.2. Characterization

The crystalline structure of the materials was analyzed by X-ray diffraction (XRD, Rigaku D/Max 2500, Toshima, Tokyo) in the 2*θ* range of 20° to 70°. The microstructure was probed via scanning electron microscopy (SEM, FEI Nova Nano 230, Ohio State University, OH, USA), transmission electron microscopy (TEM, Hitachi, H-600, Tokyo, Japan), and high-resolution transmission electron microscopy (HRTEM, JEOL JEM-2100F, Tokyo, Japan). The viscosity of ceramic inks was assessed by means of a digital viscometer (NDJ-1F, Shanghai Changji Precision Instrument, Shanghai, China). The surface tension was measured with an automatic surface tension device (DCAT9, Dataphysics Company, Stuttgart, Germany). The density of the inks was evaluated using a digital density and concentration meter (DMA 4500, Anton Parr, Graz, Austria). Zeta potentials of the inks were determined using a Zetasizer Nano ZS90 device (Malvern, Malvern, UK) at various pH values to select a suitable ceramic ink. The pH value was adjusted by mixing the ink with HCl and NH_3_·H_2_O (25 wt%) solutions and verified with a pH meter (PB-10, Sartorius, Gottingen, Germany), which was calibrated using the standard electrolytic buffer solutions with pH 5 and 11. Sedimentation was implemented via settling tests by comparing PAANH_4_ with PAA as dispersants, respectively. The inks were ultrasonically treated for 10 min and then aged in a 5 mL glass tube. The sedimentation volumes were determined and recorded artificially.

### 2.3. Printing Process

A drop-on-demand inkjet printing device system (Jetlab ΙΙ, Microlab Technologies Inc., NJ, USA) was applied to assess the printability of NiMn_2_O_4_ ceramic inks. Herein, the device mainly consisted of an X-Y-Z (600 mm × 300 mm × 100 mm) motion control module and a piezoelectric type printhead (MJ-AT-60, Microlab Technologies Inc.). The actuating voltage, pulse duration, delay, and frequency parameters of the system were adjusted as shown in Table 1. The ejection velocity of the ink droplet was about 6 m/s. The schematic illustration of the inkjet printing system is illustrated in Figure S1.

## 3. Result and Discussion

### 3.1. Structure Analysis

**Figure 1:**
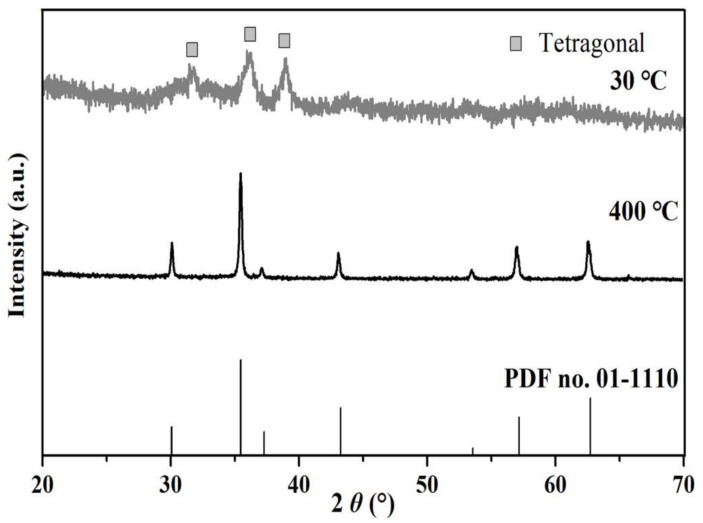
XRD pattern of the sample at 30 °C and 400 °C.

According to the XRD patterns in Figure 1, the precursor at 30 °C was mainly composed of a tetragonal spinel phase, the characteristic diffraction peaks of which corresponded to the standard card (PDF no: 18-0408). With the increase in temperature, the intensities of the diffraction peaks increased gradually, while their half-widths decreased. At the temperature of 400 °C, the crystal phase of the sample was isomorphic to cubic NiMn_2_O_4_ (PDF no.: 01-1110, space group F3dm), which indicated the diffraction peaks were mainly at 2*θ* = 30.2° (220), 35.5° (311), 37.1° (222), 43.2° (400), 53.5° (422), 57.1° (511), and 62.6° (440). Besides, no impurity phases were detected.

### 3.2. Analysis of Ceramic Powders

**Figure 2:**
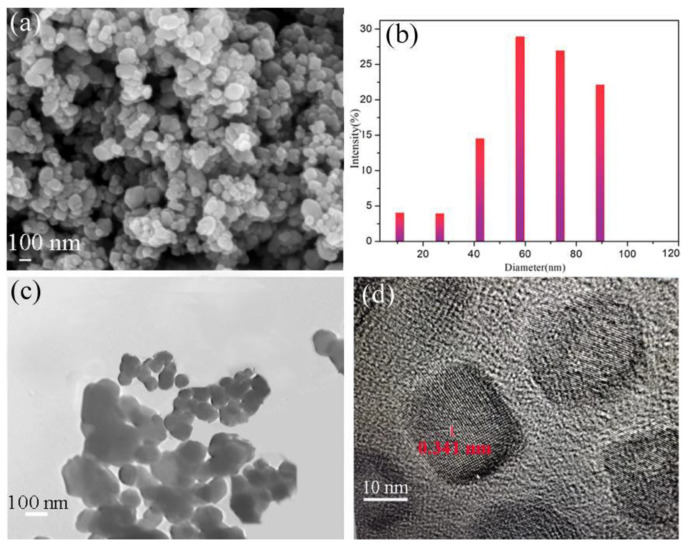
(**a**) SEM image of NiMn_2_O_4_ ceramic particle, (**b**) the particle size distributions by percent of NiMn_2_O_4_ ceramic particles, and (**c**) TEM and (**d**) HRTEM images of ceramic powders.

Figure 2a–c displays the SEM image, the particle size distribution, and the TEM image of NiMn_2_O_4_ ceramic particles. The NiMn_2_O_4_ powders showed high monodispersity and a spherical shape (Figure 2a), and the nanoparticle size ranged from 16 to 90 nm with an average of about 73 nm (Figure 2b). The HRTEM image in Figure 2d revealed the high degree of crystallinity of ceramic powders. The interplanar spacing values were measured to be about 0.341 nm and 0.265 nm, which corresponded to the (112) and (103) planes of the NiMn_2_O_4_ cubic spinel phase, respectively. The results revealed the preparation of ultra-fine (commonly smaller than 100 nm) ceramic particles by the reverse microemulsion method.

### 3.3. Ceramic Ink Characterization

**Figure 3:**
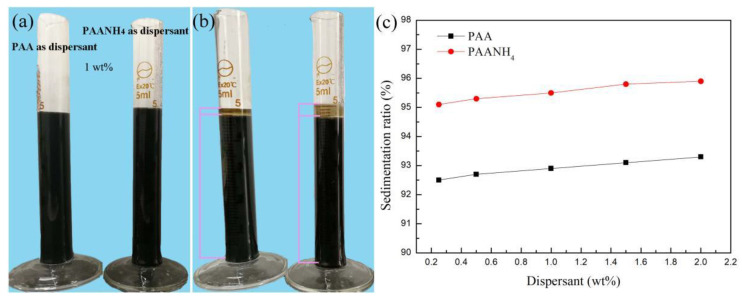
(**a**) Aging test before and (**b**) after 72 h aging of ceramic inks (solid loading 10 wt%) obtained with different dispersant PAA and PAANH_4_. (**c**) Sedimentation ratio as a function of different dispersant contents.

The results after 72 h of aging for the NiMn_2_O_4_ ceramic inks with PAA and PAANH_4_ (1 wt%) dispersants are shown in Figure 3a,b. It could be seen that the ceramic ink with PAANH_4_ had a more stable property compared with the PAA-containing one. According to [24], the dispersant content influences the rheological properties such as sedimentation ratio, as well as the printing process itself. In that regard, the sedimentation ratios were afterward calculated for each type of ceramic ink with different amounts of dispersant. The corresponding data are shown in Figure 3c, demonstrating an increase in sedimentation ratio with an increasing dispersant content. It was obvious that the PAANH_4_ dispersant had a better dispersion effect than PAA, which could be apparently observed from Figure 3a,b. Moreover, the sedimentation ratio of ceramic inks changed little relative to the dispersant content (from 92.5% to 93.1% for PAA and from 95.0% to 95.7% for PAANH_4_). Therefore, the 1 wt% PAANH_4_ dispersant was further chosen for the preparation of the NiMn_2_O_4_ ceramic ink.

**Figure 4:**
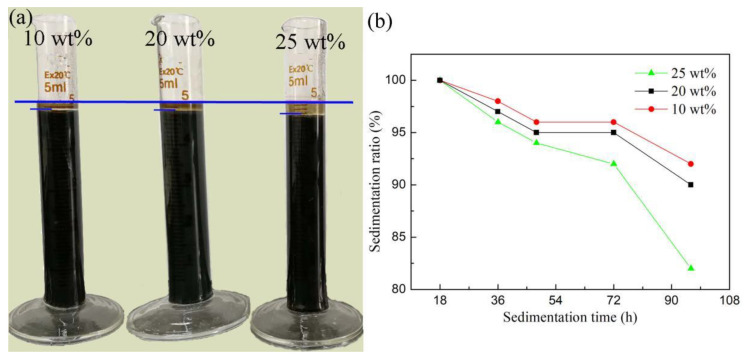
(**a**) Aging performance after 72 h of 10 wt%, 20 wt%, and 25 wt% solid loading ceramic inks obtained with dispersant PAANH_4_. (**b**) Sedimentation ratio as a function of sedimentation time for different solid content ceramic inks.

As known, high solid loading is beneficial for the densification of the printed pattern during and after solidification and sintering. As the solid content increases, the viscosity of the ceramic ink also increases, which inhibits the droplet formation [25]. The maximum solid loading is quantified from the Krieger-Dougherty equation (η=η0(1−ΦΦmax)−n), where *n* represents the empirical constant with a value of 1.8; *Φ* and *Φ*_m_ are the solid loading ratio and the maximum fraction in the ceramic ink, respectively; and *η* and *η*_0_ are the viscosities of solid-loaded and pristine ceramic inks, respectively. In the Ni-Mn-O system, the theoretical density of the powder is 5.3 g/m^3^. Given the viscosity range of (1–20) mPa·s, the theoretical maximum solid loading was calculated to be 21.3 wt%. Meanwhile, settling and aging performance of the ceramic inks with 10 wt%, 20 wt%, and 25 wt% solid loadings after 72 h are shown in Figure 4a. Compared with 10 wt% and 20 wt% inks, the 25 wt% ceramic ink displayed severe sedimentation, which was also in agreement with the above calculated value (about 21.3 wt%). Moreover, as the sedimentation time increases into 96 h, the sedimentation ratio decreases largely. For 25 wt% and 20 wt% solid content ceramic inks, the sedimentation ratio changes to 90 wt% and 82 wt%, respectively, showing the sedimentation aging property of the ceramic ink (Figure 4b). Thus, the optimal solid content in the Ni-Mn-O ceramic ink in this work was 20 wt%. Herein, a surface active agent PEG 400 with a 5.0 wt% content was afterwards used to adjust the surface tension of the ceramic ink. The ceramic ink composition for the subsequent analysis is given in Table 2.

**Figure 5:**
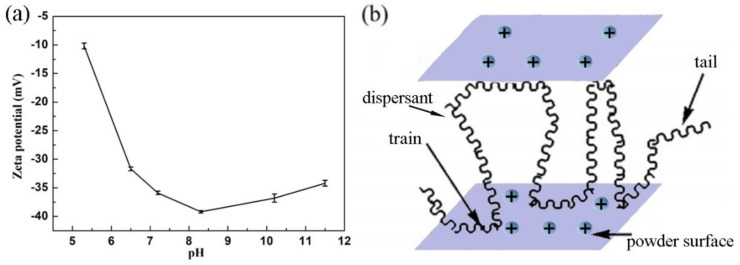
(**a**) Relationship between the zeta potential and pH value; (**b**) dispersing mechanism of the PAANH_4_.

The relationship between the zeta potential and pH value of the as-prepared 20 wt% Ni-Mn-O ceramic ink is shown in Figure 5a. The absolute value of zeta potential increased with the increasing pH value, attaining its maximum value of 40 mV at pH of 8–9. Under a low pH condition, PAANH_4_ on the ceramic particle surface produced a steric hindrance effect and could not reach a relatively stable state. With the increasing pH value, the charged carboxylic groups of PAANH_4_ led to dissociation and stretching of polymer chains containing trains and tails, and the effects of steric hindrance and electrostatic force should have caused a stronger dispersion of the 20 wt% ceramic ink [26,27,28] (see Figure 5b). Herein, a suitable ceramic ink with pH of 7.2 was chosen because of the desired zeta potential.

**Figure 6:**
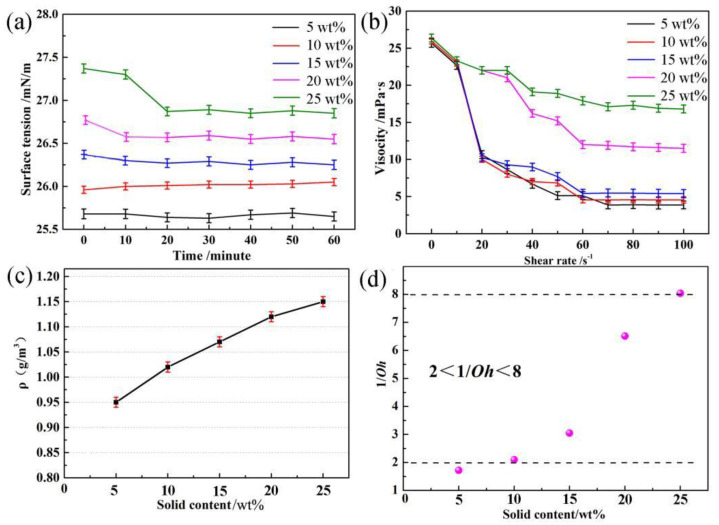
(**a**) Surface tension, (**b**) viscosity, (**c**) density, and (**d**) *Z* value of ceramic inks with different solid contents of 5 wt%, 10 wt%, 15 wt%, 20 wt%, and 25 wt%.

In order to characterize the printability of the ceramic, a quantitative analysis based on the ink’s rheological properties using the *Z* parameter as the reciprocal of dimensionless *Oh* was proposed as follows [29]:

(1) $$Z=1/Oh=\frac{R_{e}}{\sqrt{W_{e}}}=\frac{{\left(\gamma \cdot \rho \cdot a\right)}^{1/2}}{\eta }$$

where *W_e_* and *R_e_* are Weber number (*W_e_* = *v*^2^*ρa*/*γ*) and Reynolds number (*R_e_* = *vρa*/*η*), respectively; *a* represents the radius of the nozzle (60 μm); and *ρ*, *η*, and *γ* denote the density, viscosity, and surface tension of the ceramic ink. To achieve the printability, the Z value of the printable ceramic ink should be in the range of 2–8 [30]. In Figure 6a–c, the surface tension of the Ni-Mn-O ceramic inks with the solid contents from 5 to 25 wt% ranged from 25.6 to 26.8 mN/m, while the viscosity value increased from 3.7 to 17.2 mPa·s, respectively. Meanwhile, the viscosity dropped with the increasing shear rate, which indicated a typical rheological behavior of the non-Newtonian fluid [31,32,33,34]. According to Figure 6d and given the values of *a* (60 μm) and *ρ* (from 0.95 g/m^3^ to 1.15 g/m^3^), *Z* values were calculated to be 1.7, 2.1, 3.0, 6.5, and 8.1, respectively, which illustrated that the ceramic inks with the solid contents from 10 to 20 wt% were suitable for the inkjet printing process. Considering the requirement of high solid loading, the suitable 20 wt% ceramic ink was finally selected for further analysis.

**Figure 7:**
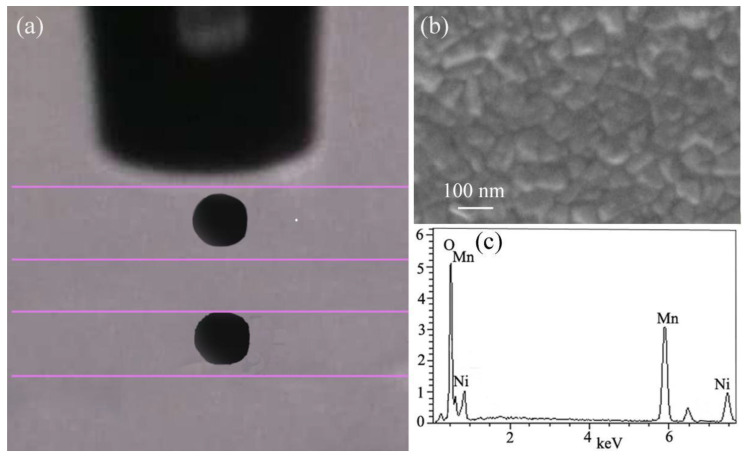
(**a**) The captured stroboscope image of the inkjet droplet with a spherical shape. (**b**) SEM image of the Ni-Mn-O film after annealing at 300 °C. (**c**) EDS spectrum of the Ni-Mn-O film.

The printable property was continuously verified using the inkjet printing system with a piezoelectric type actuator, wherein the excitation voltage of 95 V and the frequency of 1000 Hz were adjusted for the printing process [35]. The quality of inkjet printing largely depended on the rheological property of the disperse ceramic ink, which was dominated by the capillary effect, as well as the nature of capillaries, viscosity, and mobility of the ceramic ink [36]. At the initial stage of the printing, the ink was jetted from the inkjet head and then took the form of almost spherical droplets without satellites; the captured stroboscope image is shown in Figure 7a. The Ni-Mn-O ceramic droplets were steadily printed through the nozzle. The Ni-Mn-O ceramic droplets were steadily printed through the nozzle, revealing excellent printability and repeatability of the ink. Subsequently, the as-deposited Ni-Mn-O film was annealed at 300 °C for 30 min and the SEM image is shown in Figure 7b. The annealed film has a uniformity grain size of about 81.2 ± 13.5 nm, which arises from the as-prepared nanoparticles. The EDS analysis in Figure 7c shows that the annealed film consists of Mn, Ni, and O, which is in accordance with the XRD result of the sample.

## 4. Conclusions

Herein, a small amount of dispersant (PAANH_4_) was chosen and added into the as-prepared Ni-Mn-O ceramic nanopowder via the reverse microemulsion method. The settling and aging properties of the ceramic inks were tuned by adding different dispersant contents. The zeta potential, surface tension, viscosity, density, and sedimentation were measured for the as-prepared Ni-Mn-O ceramic ink with a 20 wt% solid loading. The *Z* value of theoretical printability and the properties of the ceramic ink conformed to the requirements for the inkjet system in this study. Finally, the Ni-Mn-O ceramic droplets were steadily released during in situ inkjet printing through the inkjet printhead without any satellites or tails. Therefore, the desired high solid content Ni-Mn-O ceramic ink with an excellent printing property could be obtained.

## Figures and Tables

**Table 1:** The parameters of the inkjet printing system.

Parameter	Pluse Voltage	Pluse Width	Delay Frequency	Frequency
Value	0 V, 95 V, −20 V	3 μs, 30 μs, 3 μs, 30 μs, 3 μs	150 Hz	1000 Hz

**Table 2:** The composition of Ni-Mn-O ceramic ink.

Composition	Ni-Mn-O Powder	Deionized Water	PAANH_4_	PEG 400
weight (g)	1.0	3.7	0.05	0.25
ratio (wt%)	20.0	72.0	1.0	5.0

## Data Availability

Data are available upon request.

## Supplementary Materials

The following supporting information can be downloaded at: https://www.mdpi.com/article/10.3390/ma16010021/s1, Figure S1: Schematic illustration of the ink-jet printing system.
