# Supplementary material for: Development and Characterizations of Novel Aqueous-Based Ceramic Inks for Inkjet Printing
Source: Materials (Basel). 2022 Dec 21;16(1):21. doi: 10.3390/ma16010021 (PMC9821278; doi:10.3390/ma16010021)
Supplement: Supplementary file 1 [file materials-16-00021-s001.zip › materials-2023357-supplementary.pdf]

materials MDPI

Supplementary Materials

Development and Characterizations of Novel Aqueous-Based Ceramic Inks for Inkjet Printing

The schematic illustration of the ink–jet printing system is illustrated in Figure S1.

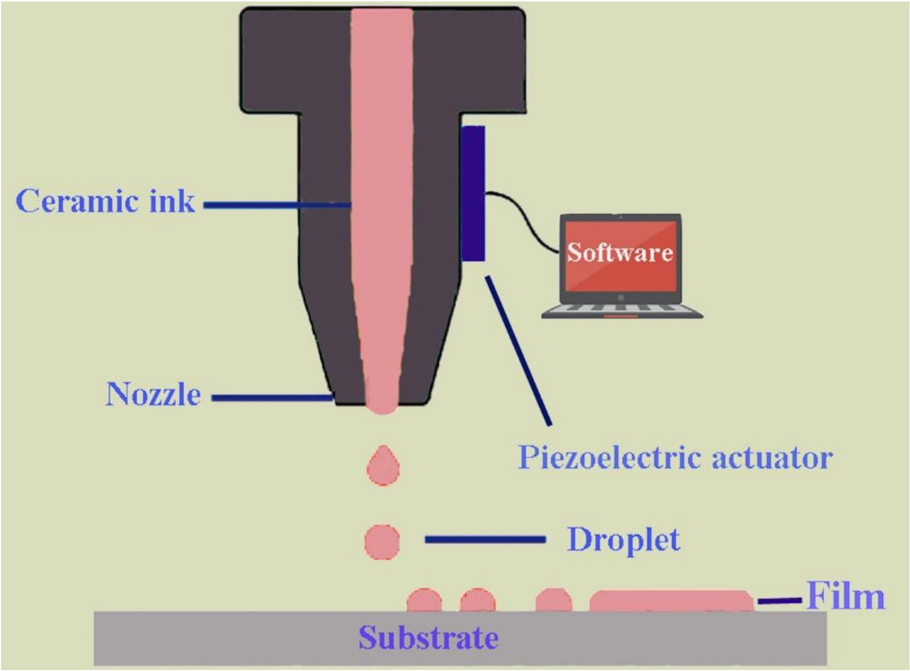

Figure S1. Schematic illustration of the ink–jet printing system.
